# Allocation Strategies for Seed Nitrogen and Phosphorus in an Alpine Meadow Along an Altitudinal Gradient on the Tibetan Plateau

**DOI:** 10.3389/fpls.2020.614644

**Published:** 2020-12-09

**Authors:** Zhiqiang Wang, Haiyan Bu, Mingcheng Wang, Heng Huang, Karl J. Niklas

**Affiliations:** ^1^Institute for Advanced Study, Chengdu University, Chengdu, China; ^2^State Key Laboratory of Grassland Agro-Ecosystems, School of Life Sciences, Lanzhou University, Lanzhou, China; ^3^Department of Environmental Science, Policy, and Management, University of California, Berkeley, Berkeley, CA, United States; ^4^Plant Biology Section, School of Integrative Plant Science, Cornell University, Ithaca, NY, United States

**Keywords:** allocation, alpine meadow, nitrogen, phosphorus, scaling exponent, seed

## Abstract

Nitrogen (N) and phosphorus (P) play important roles in many aspects of plant biology. The allocation of N and P in plant vegetative organs (i.e., leaves, stems, and fine roots) is critical to the regulation of plant growth and development. However, how these elements are allocated in seeds is unclear. The aim of this study was to explore the N and P allocation strategies of seeds in an alpine meadow along an altitudinal gradient. We measured the seed N and P contents of 253 herbaceous species in 37 families along an altitudinal gradient (2,000–4,200 m) in the east Tibetan alpine meadow. The geometric means of seed N and P concentrations and N:P ratios were 34.81 mg g^–1^, 5.06 mg g^–1^, and 6.88, respectively. Seed N and P concentrations varied across major taxonomic groups and among different altitude zones. N:P ratios showed no significant variations among different taxonomic groups with the exception of N-fixing species. The numerical value of the scaling exponent of seed N vs. P was 0.73, thus approaching 3/4, across the entire data set, but varied significantly across major taxonomic groups. In addition, the numerical value of the scaling exponent of N vs. P declined from 0.88 in the high altitude zone to 0.63 in the low altitude zone. These results indicate that the variations in the numerical value of the scaling exponent governing the seed N vs. P scaling relationship varies as a function of major taxonomic groups and among different altitude zones. We speculate that this variation reflects different adaptive strategies for survival and germination in an alpine meadow. If true, the data presented here advance our understanding of plant seed allocation strategies, and have important implications for modeling early plant growth and development.

## Introduction

Nitrogen (N) and phosphorus (P) are considered the most important resources regulating plant growth and development (Vitousek, 2004), and thus play pivotal roles in regulating community dynamics and ecosystem function (Güsewell, 2004; Vitousek et al., 2010). Nitrogen is linked with leaf photosynthesis rates and total carbohydrate storage in leaves, stems, and roots (Sterner and Elser, 2002), whereas phosphorus is directly involved in the production of DNA, RNA, and proteins (Sterner and Elser, 2002; Vrede et al., 2004). The allocation of N and P in plants therefore undoubtedly reflects fundamental adaptive strategies across different species and species groups, which in turn influence the material and energy cycles of ecosystems (Ågren, 2004; Elser et al., 2007). Thus, quantifying how these two elements are allocated in plants is essential to our understanding of ecosystem dynamics.

The allocation patterns of N and P in the major vegetative plant organs (i.e., leaves, stems, and fine roots) are of particular interest and have been extensively investigated. Prior work has shown that the N vs. P scaling relationship is described by a power function taking the form of N = βP^α^, where β and α represent the normalization constant and the slope (i.e., the scaling exponent) of the log-log linear N vs. P regression curve, respectively (Wright et al., 2004; Niklas et al., 2005; Kerkhoff et al., 2006; Niklas and Cobb, 2006b). The scaling exponent (α) is a crucial parameter in any power function. In the context of the N vs. P scaling relationship, when the scaling exponent is less than unity (i.e., α < 1), plants require disproportionately more P than N, perhaps to ensure rapid protein synthesis (as predicted by the growth rate hypothesis proposed by Elser et al., 2000; 2003), whereas when the scaling exponent is greater than unity (i.e., α > 1) the opposite trend holds true (McGroddy et al., 2004; Niklas et al., 2005; Kerkhoff et al., 2006).

It has been observed that the allocation of N and P in plant organs is consistent with fundamental stoichiometric theory (Wright et al., 2004; Kerkhoff et al., 2006; Reich et al., 2010), and some comprehensive studies have reported that the scaling exponent of the N vs. P scaling relationship is numerically constant across independently evolved lineages despite environmental differences. Nevertheless, there remains a debate as to the numerical value of the scaling exponent. For example, using extensive data sets for leaves, 2/3 and 3/4-power “laws” have been proposed by different workers (Reich and Oleksyn, 2004; Wright et al., 2004; Niklas, 2006a; Reich et al., 2010). Furthermore, based on 1890 observations of 763 terrestrial plant species, Wang et al. (2019) have proposed a 0.82-power “law” for global fine root N vs. P across different plant groups and ecosystems. Consequently, the scaling exponent for the N vs. P scaling relationship manifests statistically significant differences between leaves and fine roots. However, several studies have also reported N vs. P scaling exponents with statistically significantly different numerical values in leaves (McGroddy et al., 2004; Han et al., 2005; Niklas et al., 2005; Tian et al., 2018), stems (Kerkhoff et al., 2006; Wang et al., 2018; Zhang et al., 2018) and fine roots (Yuan et al., 2011; Geng et al., 2014; Zhao et al., 2016) for different taxonomic groups or geographical locations owing to different physiological growth strategies among species and responses to changes in the environment.

In contrast to studies that have focused on leaves, stems, or fine roots, comparatively little is known about how N and P are allocated in seeds, which play an important role in the plant life cycle and directly influence the perseverance of species in different environmental settings (Bewley, 1997; Jiménez-Alfaro et al., 2016). During the earlier stages of ontogeny, a series of morphological and physiological processes depend largely on seed reserves (i.e., endosperm or cotyledons; see Muthukumar and Udaiyan, 2000). N and P reserves in seeds supply critical components for the synthesis of ribosomes, RNA, DNA, and proteins required for cell division, embryonic growth and development, and seedling establishment (Bewley, 1997; Slot et al., 2013; Bu et al., 2016). Given the importance of seed N and P in early growth and survival, it is important to understand and quantify the N vs. P scaling relationship in seeds.

Previous studies have demonstrated that the N and P concentrations in seeds are influenced by many environmental factors, such as temperature (Xu et al., 2016), CO_2_ (He et al., 2005), light (Mathew et al., 2000) and latitude (de Frenne et al., 2011) that can change as a function of altitude. Consequently, environmental variations can exert selective pressures on seed mass, stock nutrient materials, and germination time, thereby resulting in different adaptive strategies (Lord, 1994). For example, Bu et al. (2018) found that altitude has a significant effect on N and P seed concentrations. Based on this limited information, we hypothesized that the N vs. P scaling relationship for seeds will vary as a function of altitude.

The Tibetan Plateau represents one of the largest alpine meadows in the world, wherein herbaceous species have adapted to high altitude and low temperature by evolving unique survival mechanisms such as small seed size (Turnbull et al., 2012), rapid growth and life history completion (Adler et al., 2014), and high nutrient concentrations (Körner, 1989). Thus, the Tibetan Plateau provides an ideal region to investigate the N and P allocation strategies of seeds. For this purpose, we compiled a dataset on paired measurements of N and P seed concentrations for herbaceous species growing along an altitudinal gradient (2,000–4,200 m) on the east Tibetan Plateau. These data were then used to answer three important questions: (1) do seed N and P concentrations and N:P ratios differ across major taxonomic groups and among different altitude zones?, (2) is the seed N vs. P scaling relationship the same across major taxonomic groups?, and (3) does the numerical value of the scaling exponent governing N vs. P relationships change along an altitudinal gradient?

## Materials and Methods

### Study Site

**FIGURE 1:**
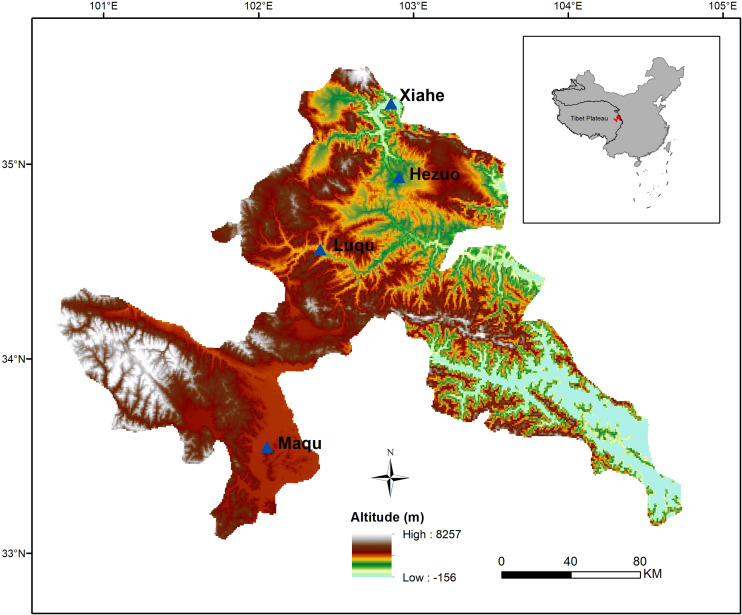
The study area and sample collection sites.

This study was conducted on the northeastern edge of the Tibetan Plateau in China (100°44′–104°45′E, 33°06′–35°34′, altitude: 2,000–4,200 m) (Figure 1). The main vegetation is that of a typical alpine meadow. The climate is cold Humid-Alpine (annual average temperature ranges from 1.2°C to 4.6°C and annual average precipitation ranges from 516 mm to 780 mm), and growing season is short (from late May to late September) (Bu et al., 2018, 2019). The dominant species in alpine meadow are mainly from the Asteraceae, Cyperaceae, Fabaceae, Gentianaceae, Polygonaceae, Ranunculaceae, and Scrophulariaceae (Zhang et al., 2014).

### Seed Sampling and Measurement

From late August to September in 2015, mature seeds of 253 species from 37 families were collected from four zones along an altitudinal gradient (2,000–4,200 m). The time mature seeds were collected in different altitude zones based on extensive field observations on seed development and dispersal. Mature seeds were collected randomly from more than 30 individual conspecifics for each species from sites differing in 50 m across the altitude gradient, which was divided into four equal zones (see below).

Seeds were subsequently oven-dried at 50°C to a constant mass and ground into a fine powder. N and P concentrations were measured by dry combustion on an elemental analyzer (Elementar TOC Vario, Germany) and the molybdenum blue method on an automatic flow injection analyzer (Lachat Quickchem 8500, United States), respectively, as described by Bu et al. (2018). In total, the data set included 751 paired observations of N and P for seeds.

### Data Analysis

The data were sorted into two life form groups (i.e., annual and perennial), two functional groups (i.e., forb and graminoid), two N-fixing groups (i.e., N-fixing and non-N-fixing), and two phylogenetic groups (i.e., monocotyledon and dicotyledon). The data were also divided into four altitude zones: Maqu (3,400–4,200 m), Luqu (3,000–3,400 m), Hezuo (2,600–3,000 m) and Xiahe (2,000–2,600 m).

Differences in N and P concentrations and N:P ratios across major taxonomic groups and among the four altitude zones were assessed using one-way analysis of variance (ANOVA) with least-significant difference (LSD) *post hoc* tests. Reduced major axis (RMA) regression using the lodel2 function in ‘lmodel2’ package was used to determine the numerical values of N vs. P scaling exponents for the major taxonomic groups and altitude zones using log_10_-transformed values of N and P concentrations (Warton et al., 2006; Legendre, 2018). A likelihood-ratio test method was used to assess the heterogeneity of RMA scaling exponents within the aforementioned groupings and altitude zones. All statistical analyses were performed using SPSS v20 (SPSS Inc., United States) and the statistical software R 2.15.2 (R Development Core Team, 2015).

## Results

**FIGURE 2:**
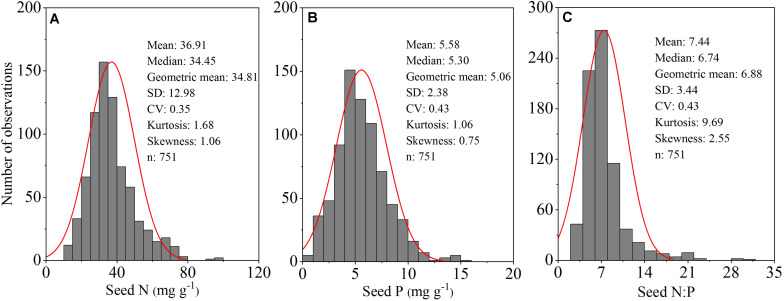
Frequency distribution of seed nitrogen (N) (mg g^–1^) **(A)**; phosphorus (P) (mg g^–1^) **(B)** and N:P ratios **(C)** for all 253 herbaceous species in an alpine meadow on the northeast Tibetan Plateau.

We found that N and P concentrations differed across major taxonomic groups (Table 1 and Figure 2). For example, N concentrations ranged from 22.62 mg g^–1^ for graminoids to 57.08 mg g^–1^ for N-fixing species, whereas P concentrations ranged from 3.17 mg g^–1^ for graminoids to 5.21 mg g^–1^ for dicotyledon. In contrast, N:P ratios did not show significant difference, except for the two N-fixing groups. N-fixers had higher N:P ratios (11.33) than non-fixers (6.61). Furthermore, the geometric mean values of N and P concentrations and N:P ratios among the four altitude zones were confined to a relatively modest numerical range. Specifically, the N concentration ranged from 32.17 mg g^–1^ in Luqu to 37.67 mg g^–1^ in Maqu, whereas the P concentration ranged from 4.60 mg g^–1^ in Xiahe to 5.59 mg g^–1^ in Maqu; N:P ratios ranged from 6.71 in Hezuo to 7.33 in Xiahe (Table 1).

**TABLE 1:** Geometric means of seed N, P concentrations and N:P ratios for major taxonomic groups and altitude zones.

Taxonomic group	*n*	N	P	N:P
All	751	34.81 (0.47)	5.06 (0.09)	6.88 (0.13)
*Life form groups*				
Annual	188	31.03 (0.60)b	4.86 (0.16)a	6.38 (0.31)a
Perennial	563	36.17 (0.58)a	5.12 (0.10)a	7.06 (0.13)a
*Functional groups*				
Forb	714	35.56 (0.48)a	5.18 (0.09)a	6.87 (0.13)a
Graminoid	37	22.62 (1.57)b	3.17 (0.33)b	7.15 (0.60)a
*N-fixation groups*				
N-fixing	57	57.08 (1.75)a	5.04 (0.28)a	11.33 (0.61)a
Non-N-fixing	694	33.42 (0.43)b	5.06 (0.09)a	6.61 (0.12)b
*Phylogeny groups*				
Monocotyledon	59	26.34 (1.53)b	3.58 (0.21)b	7.40 (0.41)a
Dicotyledon	692	35.64 (0.49)a	5.21 (0.09)a	6.84 (0.13)a
*Sites*				
Maqu	277	37.67 (0.81)a	5.59 (0.14)a	6.74 (0.13)b
Luqu	188	32.17 (0.84)b	4.68 (0.16)b	6.88 (0.27)b
Hezuo	137	34.18 (1.26)b	5.10 (0.23)a	6.71 (0.40)b
Xiahe	149	33.75 (0.91)b	4.60 (0.19)b	7.33 (0.29)a

**n* represents the number of samples. SE in brackets is the standard error. Different letters denote significant differences at the 0.05 level.*

**FIGURE 3:**
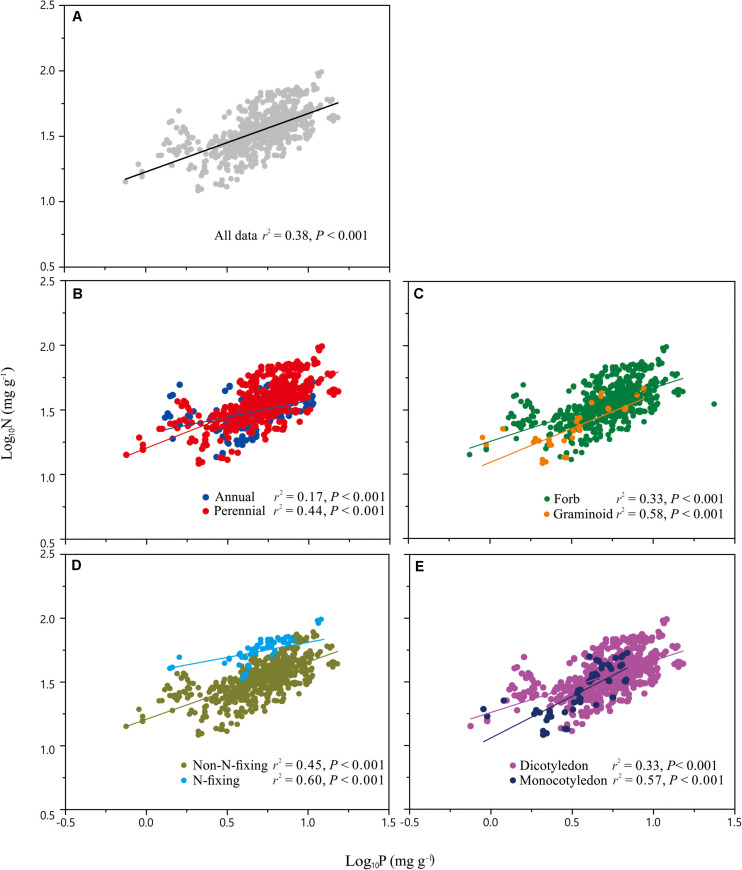
Scaling relationships of seed nitrogen (N) vs. phosphorus (P) for the entire data set **(A)**, and for plants in two life form groups (annual, perennial) **(B)**, two functional groups (forb, graminoid) **(C)**, two N-fixation groups (N-fixing, non-N-fixing) **(D)** and two phylogeny groups (monocotyledon, dicotyledon) **(E)**. Statistical parameters for reduced major axis (RMA) regressions are provided in Table 2.

**FIGURE 4:**
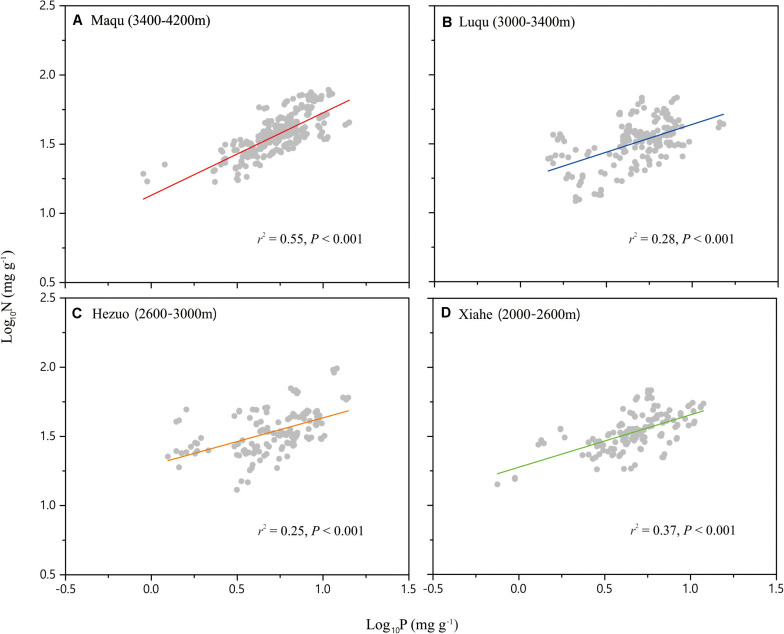
Scaling relationships of seed nitrogen (N) vs. phosphorus (P) in an alpine meadow for the four altitude zones: Maqu **(A)**, Luqu **(B)**, Hezuo **(C),** and Xiahe **(D)**. Statistical parameters for reduced major axis (RMA) regressions are provided in Table 3.

Across the entire dataset, the numerical value of the N vs. P scaling exponent was 0.73 (*n* = 751, 95% CIs = 0.69–0.77, *r*^2^ = 0.38, *P* < 0.001) (Figure 3). In the case of the taxonomic groups, the numerical values of scaling exponents varied significantly (*P* < 0.001) among annuals (0.60), perennials (0.75), N-fixers (0.54), non-N-fixers (0.67), monocots (0.87), and dicots (0.71) (Table 2). However, the numerical values of the scaling exponent for forbs (0.72) and graminoids (0.69) showed no significant difference (*P* > 0.05). Moreover, the scaling exponents for N vs. P numerically differed along the altitudinal gradient (Table 3 and Figure 4), e.g., the scaling exponent decreased from 0.88 in the high altitude Maqu zone to 0.63 in the low altitude Xiahe zone.

**TABLE 2:** Summary of reduced major axis (RMA) regression statistics for seed N vs. seed P concentrations for different taxonomic groups (all relations are significant at *P* < 0.001).

Taxonomic group	*n*	Intercept	Exponent	Low CI	High CI	*r*^2^
All	751	1.03	0.73	0.69	0.77	0.38
*Life form groups*						
Annual	188	1.08	0.60b	0.52	0.58	0.17
Perennial	563	1.03	0.75a	0.7	0.79	0.44
*Functional groups*						
Forb	714	1.04	0.72a	0.68	0.76	0.33
Graminoid	37	1.01	0.69a	0.54	0.85	0.58
*N-fixation groups*						
N-fixing	57	1.38	0.54b	0.45	0.63	0.60
Non-N-fixing	694	1.05	0.67a	0.63	0.71	0.45
*Phylogeny groups*						
Monocotyledon	59	0.94	0.87a	0.85	1.03	0.57
Dicotyledon	692	1.04	0.71b	0.67	0.75	0.33

**n* represent the number of observations, and letters denote significant differences in exponents based on a likelihood ratio test. All data were log_10_-transformed for analysis.*

**TABLE 3:** Summary of reduced major axis (RMA) regression statistics for seed N vs. seed P concentrations for four altitudinal zones (all relations are statistically significant at *P* < 0.001).

Site	Altitude	*n*	Intercept	Exponent	Low CI	High CI	*r*^2^
Maqu	3400–4200	277	0.98	0.88a	0.74	0.87	0.55
Luqu	3000–3400	188	1.00	0.76b	0.67	0.85	0.28
Hezuo	2600–3000	137	1.05	0.68c	0.58	0.78	0.25
Xiahe	2000–2600	149	1.11	0.63c	0.55	0.71	0.37

*Letters denote significant differences in exponents based on a likelihood ratio test. All data were log_10_-transformed for analysis.*

## Discussion

### N, P Concentrations and N:P Ratios in Seeds

The data presented here indicate that the arithmetic mean values of seed N and P concentrations (36.91 and 5.58 mg g^–1^) are higher than leaves (29.20 and 2.0 mg g^–1^) reported by He et al. (2008) and fine roots (11.09 and 0.91 mg g^–1^) reported by Geng et al. (2014) for herbaceous species in an alpine meadow. This finding is consistent with the fact that germinating seeds manifest relatively higher metabolic activity and require more nutrient resources for rapid germination compared to leaves or fine roots (Bu et al., 2008) until seedlings become autotrophic and established during a short growing season (Soriano et al., 2011; Bewley, 1997; Bewley et al., 2013). In this respect, the data support the growth rate hypothesis, i.e., fast-growing herbaceous species have higher N and P concentrations and lower N:P ratios (Sterner and Elser, 2002).

Seed N and P concentrations varied across major taxonomic groups (Table 1). These differences may simply reflect divergence in nutrient use and metabolic strategies. For example, higher N concentrations are observed for perennial species compared to annual species. This result is in agreement with the study of Bu et al. (2008) who suggest that higher seed N content is positively correlated with germination success. The seeds of N-fixing species have significantly higher N concentrations than non-N-fixing species owing to their ability to absorb N in fine roots (McCormack et al., 2015) and also perhaps because of the high metabolic cost of N-fixation (Wardle and Greenfield, 1991). Higher N and P concentrations occur in forbs than in graminoids, supporting the idea that seeds of forbs tend to germinate more rapidly and produce more vigorous seedlings compared to the seeds of grasses (Ching and Rynd, 1978). Similarly, Dicotyledon tend to have higher N and P concentrations than monocotyledon, perhaps as a result of differences in how nutrient reserves are stored (Bonfil, 1998). The N:P ratios of the seeds collected in this study do not vary across major taxonomic groups, with the exception of N-fixers. The relative constancy of seed N:P ratios across major taxonomic groups may reflect a fundamental constraint on seed N and P stoichiometry.

Finally, variations in seed N and P concentrations and N:P ratios among the four altitude zones are statistically discernable. The high N and P concentrations in the Maqu zone were significantly higher than those in the other three regions. This may reflect an adaptive strategy to deal with low temperatures and a very short growing season (Bu et al., 2016). Under any circumstances, our data support the temperature-plant physiological hypothesis (Reich and Oleksyn, 2004), which states that higher N and P concentrations at low temperatures offset reductions in metabolic reaction rates and enhance cold hardiness.

### The N vs. P Scaling Exponent Across Major Taxonomic Groups

The analysis of the pooled data set indicates that the numerical value of the scaling exponent governing the seed N vs. P scaling relationship is 0.73. This value is consistent with 3/4-power “rule” reported for leaves (Reich and Oleksyn, 2004; Niklas, 2006a), and is significantly different from the numerical values reported by other workers for leaves (Wright et al., 2004; Reich et al., 2010; Tian et al., 2018) or for fine roots (Wang et al., 2019). The observed 3/4-power “rule” for seeds indicates that P concentrations increase faster than N concentrations (which may reflect the fact that protein synthesis during early seed germination primarily relies on mRNA reserves which are P-rich), which once again supports the growth rate hypothesis of Sterner and Elser (2002). In addition, this value is inconsistent with those of Kerkhoff et al. (2006), who found that the N vs. P scaling exponent in reproductive structures is close to 1. However, it must be noted that, compared to previous studies using extensive global data sets for leaves and fine roots, our data come only from herbaceous species growing in an alpine meadow.

Although more data for different species and biomes are required to determine whether the seed N vs. P scaling exponent interspecifically converges onto a “canonical” value, it is clear that the numerical values of the scaling exponent differs significantly among contrasting taxonomic groups (Table 2, Figure 3). For example, compared to annual species, perennial species have numerically higher N vs. P scaling exponents (0.60 vs. 0.75), suggesting that the short life cycle of annual species requires rapid growth rates. The N vs. P scaling exponent for N-fixing species (0.54) is lower than non-N-fixing species (0.67), indicating that N-fixing species with higher N:P ratios may have a competitive advantage for P absorption over non-N-fixing species. The numerically smaller N vs. P scaling exponent observed for dicotyledon (0.71) relative to monocotyledon (0.87) might reflect differences in the ways nutrients are stored and provided (i.e., storage in cotyledons vs. endosperm), thereby providing different nutrient allocation strategies resulting from evolutionary selection pressures on plant physiology (He et al., 2006). However, in the case of the two functional groups, the numerical value of scaling exponents did not differ between forbs and graminoids. In this context, it is also important to note that the scaling exponents reported here for graminoids have broad 95% CIs (i.e., 0.85 and 0.54) which include the 0.72 scaling exponent observed for forbs. Therefore, more data from graminoids are needed.

### N vs. P Scaling Exponent Along the Altitudinal Gradient

The numerical value of the seed N vs. P scaling exponent varied significantly and declined from the high altitude Maqu zone (0.88) to the low altitude Xiahe zone (0.63) (Table 3, Figure 4). We suggest that this phenomenology reflects different strategies to cope with lower temperatures and shorter growing seasons with increasing altitude (Bazzaz and Grace, 1997). In Xiahe, the relatively high growing season temperature and length and high soil N and P availability can promote rapid germination. In contrast, with increasing altitude, lower temperatures can depress the absorption of soil nutrients, thereby reduce in the availability of N and P during germination (Aerts and Chapin, 2000).

## Conclusion

In summary, this study shows that the numerical value of the seed N vs. P scaling exponent differs across major taxonomic groups and declines from high to low altitude. Based on these data, we suggest that herbaceous species in alpine meadow allocate N and P in seeds to maximize germination and seedling success in response to differences in their environment. These results advance our understanding of plant seed allocation strategies, and have important implications for modeling early plant growth.

## Data Availability Statement

The original contributions presented in the study are included in the article/supplementary materials, further inquiries can be directed to the corresponding author/s.

## Author Contributions

ZW, HH, and KN conceived the idea and designed the research. ZW, MW, and HB collected the data. All authors performed the data analysis, wrote the manuscript and gave final approval for publication.

## Conflict of Interest

The authors declare that the research was conducted in the absence of any commercial or financial relationships that could be construed as a potential conflict of interest.
